# Suicidal and self-harm behaviors in emergency psychiatry: bridging risk assessment and clinical decision-making

**DOI:** 10.3389/fpsyt.2026.1843176

**Published:** 2026-06-24

**Authors:** Íñigo Alberdi-Páramo, Irene Rodrigo-Holgado, Marina Díaz-Marsá

**Affiliations:** 1 Psychiatrist, Department of Psychiatry and Mental Health, Hospital Clinico San Carlos, Madrid, Spain; 2 Associate Professor of Health Sciences, Faculty of Medicine, Complutense University of Madrid, Madrid, Spain; 3 Clinical Psychologist, Department of Psychiatry and Mental Health, Hospital Universitario 12 de Octubre, Madrid, Spain

**Keywords:** clinical decision-making, crisis intervention, emergency psychiatry, non-suicidal self-injury (NSSI), self-harm, suicide, suicide risk assessment

## Abstract

**Background:**

Suicidal behavior and non-suicidal self-injury (NSSI) represent major clinical challenges in psychiatric emergency settings. Despite extensive research on risk factors, the prediction and management of suicidal behavior remain complex, often requiring rapid decision-making under conditions of uncertainty.

**Objective:**

To provide a practical, clinically grounded framework for the assessment and management of suicidal and self-harm behaviors in emergency settings, integrating current evidence with real-world clinical practice.

**Methods:**

This narrative review synthesizes key findings from the literature alongside clinical experience in psychiatric emergency care. Core domains include conceptual definitions, functions of self-harm, suicide risk assessment, and acute management strategies.

**Results:**

Self-harm behaviors frequently serve multiple intrapersonal and interpersonal functions, including emotional regulation, anti-dissociative mechanisms, and crisis communication. Suicide risk assessment should move beyond static risk factor models and incorporate dynamic elements such as intent, planning, ambivalence, and protective factors, including reasons for living. In emergency settings, management should prioritize therapeutic engagement, emotional validation, and collaborative decision-making. The distinction between acute and chronic risk is critical for appropriate disposition, including hospitalization versus outpatient management.

**Conclusions:**

Effective management of suicidal and self-harm behaviors in emergency contexts requires an integrative, patient-centered approach that combines structured assessment with clinical judgment. Emphasis on therapeutic alliance and individualized care planning may improve outcomes beyond traditional risk stratification models.

## Highlights

- Suicide risk assessment should move beyond predictive models toward clinical formulation
- Self-harm behaviors require a functional understanding
- Emergency management should prioritize therapeutic engagement
- Disposition decisions should integrate risk, context, and collaboration

## 1 Introduction

Suicidal behavior and non-suicidal self-injury (NSSI) represent major public health concerns and remain among the most challenging presentations in psychiatric practice (1, 2). Emergency departments frequently constitute the primary point of contact for individuals experiencing acute suicidal crises or engaging in self-harm behaviors, placing clinicians in a position that requires rapid assessment and high-stakes decision-making (3, 4). Although these settings are not always ideal for comprehensive psychiatric evaluation, they often serve as the critical gateway to care and intervention (5–7). This review focuses primarily on adult patients assessed in psychiatric emergency settings, although several principles may also be relevant to general emergency care.

Self-harm and suicidal behaviors exist along a clinical continuum that ranges from passive death wishes and suicidal ideation to suicide attempts and death by suicide (8). Importantly, NSSI may function as the “visible tip of the iceberg,” concealing underlying suicidal ideation or emerging plans (9). This conceptualization underscores the prognostic and clinical relevance of self-harm behaviors, which are consistently associated with increased risk of future suicide attempts and poorer functional outcomes across psychiatric disorders (10).

Despite decades of research, the prediction of suicidal behavior remains limited. Traditional models based on static risk factors—such as prior attempts, psychiatric diagnosis, or demographic variables—have demonstrated insufficient predictive validity at the individual level (11, 12). Consequently, there is increasing recognition that suicide risk assessment must move beyond checklist-based approaches and incorporate dynamic, context-sensitive elements, including current intent, planning, ambivalence, emotional states, and the individual’s relationship to life and death (13, 14). In this regard, protective factors—particularly personal reasons for living and social connectedness—play a crucial yet often underemphasized role in clinical evaluation (15, 16).

In parallel, NSSI has been conceptualized as a multifaceted behavior serving intrapersonal and interpersonal functions (17, 18). These include emotional regulation, relief from dissociative states, self-punishment, and communication of distress (19, 20). Understanding these functions is essential, as it reframes self-harm not merely as a risk marker but as a maladaptive coping strategy that requires targeted clinical intervention.

The management of suicidal and self-harm behaviors in emergency settings presents additional complexities (21–23). Clinicians must balance the need for safety with the preservation of patient autonomy, while establishing a therapeutic alliance under time constraints and often in emotionally charged situations (24, 25). Furthermore, decisions regarding hospitalization versus outpatient management depend not only on assessed suicide risk, but also on contextual factors such as social support, treatment adherence, and the patient’s capacity for collaboration (5, 22).

Given these challenges, there is a need for clinically oriented frameworks that integrate empirical evidence with real-world practice. The aim of this narrative review is to provide a practical and structured approach to the assessment and management of suicidal and self-harm behaviors in emergency psychiatry. By bridging existing knowledge with clinical reasoning, this work seeks to support decision-making processes and promote more effective, patient-centered care in acute settings.

## 2 Methodological approach

This article was developed as a clinically oriented narrative review integrating evidence from the literature with the authors’ experience in psychiatric emergency care. Literature was identified through non-systematic searches in PubMed and related databases focusing on suicidal behavior, non-suicidal self-injury, emergency psychiatry, crisis intervention, and suicide risk assessment.

Priority was given to influential reviews, clinical guidelines, meta-analyses, and studies with direct relevance to emergency psychiatric practice. The aim of this review was not to provide exhaustive systematic coverage, but rather to synthesize clinically meaningful concepts and approaches applicable to real-world decision-making.

## 3 Conceptualization of suicidal and self-harm behaviors

Clear and consistent terminology is essential for both clinical practice and research in the field of suicidal behavior and self-harm (2, 26). However, variability in definitions has historically led to confusion, particularly when distinguishing between suicidal and non-suicidal phenomena (18). Establishing precise conceptual boundaries is therefore a necessary first step for accurate assessment and appropriate intervention (27).

Suicidal behavior is best understood as a spectrum that includes suicidal ideation, suicide planning, suicide attempts, and death by suicide (2, 7, 20). Suicidal ideation refers to thoughts about ending one’s life, which may range from passive wishes (e.g., a desire not to wake up) to active thoughts involving intent to act (28). Suicide planning generally refers to the development of a more organized or elaborated strategy for a suicide attempt, which may include consideration of method, timing, location, access to means, and preparatory actions. However, there is no universally agreed-upon definition of suicide planning, and conceptualizations may vary across studies and clinical contexts (29). Suicide attempts refer to self-injurious behaviors carried out with at least some degree of intent to die (8). These distinctions are clinically relevant, as they reflect increasing levels of risk and require different management strategies (7).

In contrast, NSSI is defined as the deliberate destruction of body tissue without conscious suicidal intent (20). Common examples include cutting, burning, or scratching the skin (30, 31). Although the absence of intent to die differentiates NSSI from suicide attempts, this distinction is not always straightforward in clinical practice (31–33). Patients may present with ambivalence, fluctuating intent, or limited insight into their motivations, highlighting the importance of careful and nuanced assessment (20). The main functions of NSSI are summarized in **Table 1**.

**Table 1:** Main functions of non-suicidal self-injury.

Function	Description
Emotional regulation	Reduction of intense negative affect or emotional overload
Anti-dissociative	Restoration of emotional or bodily awareness
Anti-suicidal	Temporary reduction of suicidal ideation
Interpersonal influence	Communication of distress or elicitation of care
Interpersonal boundaries	Assertion of autonomy or differentiation from others
Self-punishment	Expression of guilt, shame, or self-directed anger
Sensation-seeking	Generation of feelings in states of numbness or emptiness

Understanding the function of self-harm has direct clinical implications. It shifts the focus from the behavior itself to the underlying needs it attempts to address, thereby informing more targeted interventions. In emergency settings, even brief exploration of these functions can enhance therapeutic engagement, reduce misinterpretation of intent, and guide immediate management strategies. Importantly, adopting a functional approach may also mitigate negative countertransference reactions among clinicians, fostering a more empathic and collaborative stance.

The relationship between NSSI and suicidal behavior is complex and dynamic (18). While NSSI is conceptually distinct from suicidal acts, it is strongly associated with increased risk of future suicide attempts (9). Several models have suggested that repeated exposure to self-injury may reduce fear of pain and death, thereby facilitating the transition from ideation to action (19). At the same time, NSSI may temporarily reduce suicidal urges in some individuals by regulating overwhelming emotional states, although this effect is typically short-term and does not reduce long-term suicide risk (20). Importantly, the presence of NSSI remains strongly associated with increased risk of future suicidal ideation, suicide attempts, and suicide-related morbidity.

Additional constructs further complicate the clinical picture. Suicide threats or gestures refer to behaviors intended to communicate distress or influence others without a genuine intention to die, although their predictive value should not be underestimated (1, 2). Similarly, preparatory behaviors—such as writing a suicide note, organizing personal affairs, or acquiring means—may signal escalating risk even in the absence of a recent attempt (7, 34).

Given this complexity, it is crucial to move beyond rigid categorical distinctions and adopt a dimensional and functional perspective. Rather than focusing exclusively on the presence or absence of suicidal intent, clinicians should explore the meaning, function, and context of each behavior (35, 36). This approach allows for a more accurate understanding of the patient’s internal experience and supports more individualized and effective clinical decision-making (10).

## 4 Epidemiology and clinical significance

Suicidal behavior represents a major global health burden, with suicide ranking among the leading causes of death worldwide (1). According to the World Health Organization, over 700,000 people die by suicide each year globally, making it one of the leading causes of death worldwide (33). Beyond death by suicide, non-fatal suicidal behaviors and NSSI are substantially more prevalent and account for a significant proportion of psychiatric morbidity and healthcare utilization (9). These phenomena are particularly prominent in emergency settings, where they constitute a frequent reason for psychiatric consultation (4).

The incidence of self-harm has increased substantially in recent years, particularly among adolescents and young adults, with several studies reporting marked rises in emergency department presentations related to self-injury and suicidal crises (37). This rise underscores the need for improved assessment and intervention strategies in acute care settings, where early detection may alter clinical trajectories (5, 22).

From a prognostic perspective, both suicide attempts and NSSI are among the strongest predictors of future suicidal behavior (1, 38, 39). Individuals presenting to emergency departments with self-harm have a markedly elevated risk of subsequent suicide, particularly within the first months following the index episode (4, 7). This period represents a critical window for intervention, yet it is often characterized by fragmentation of care and limited continuity between emergency and outpatient services (3, 22).

In addition to mortality risk, self-harm behaviors are associated with substantial functional impairment, psychiatric comorbidity, and reduced quality of life (1). They frequently co-occur with mood disorders, substance use disorders, personality disorders, and trauma-related conditions, further complicating assessment and management (8). Importantly, the clinical significance of these behaviors extends beyond diagnostic categories, as they often reflect underlying difficulties in emotional regulation, interpersonal functioning, and stress adaptation (2, 40).

Emergency departments occupy a pivotal role in this context. Although not designed for longitudinal care, they represent a key opportunity for engagement, risk assessment, and initiation of therapeutic pathways. The high frequency and clinical relevance of suicidal and self-harm presentations in these settings highlight the need for structured, evidence-informed approaches that can be feasibly implemented in time-constrained environments (3–5, 22).

## 5 Suicide risk assessment

### 5.1 Limitations of traditional risk prediction models

Suicide risk assessment remains a central yet inherently complex component of psychiatric evaluation (28). Despite extensive research, the ability to predict suicidal behavior at the individual level remains limited (41, 42). Traditional approaches have largely relied on the identification of static risk factors—such as prior suicide attempts, psychiatric diagnoses, substance use, or demographic variables (28, 43). While these factors are consistently associated with increased risk at a population level, their predictive value in individual cases is modest (44, 45).

Structured tools and risk scales have been developed to support clinical decision-making; however, none have demonstrated sufficient sensitivity and specificity to reliably predict suicide (46). As a result, overreliance on checklist-based approaches may create a false sense of precision and obscure the nuanced, dynamic nature of suicidal crises (47, 48). Contemporary perspectives increasingly emphasize that suicide risk assessment should not be reduced to a scoring system, but rather understood as a process of clinical formulation (13, 42, 43). Additionally, suicide risk assessment often relies on self-report, which may be limited by underreporting, ambivalence, or fluctuating intent (49).

### 5.2 Core components of a comprehensive assessment

A clinically meaningful assessment requires a multidimensional approach that integrates both objective and subjective elements (19). At its core, suicide risk assessment should include a detailed exploration of suicidal ideation, intent, planning, and behavior (45, 50).

Clinicians should assess the presence, frequency, and intensity of suicidal thoughts, as well as the degree of control the individual perceives over them. The progression from passive death wishes to active ideation and planning is particularly relevant, as it reflects increasing levels of risk (51). Exploration of preparatory behaviors—such as acquiring means, writing farewell notes, or organizing personal affairs—provides additional insight into the immediacy and seriousness of intent (38).

Equally important is the evaluation of the individual’s attitude toward suicidal thoughts and behaviors (52). Ambivalence is a common and clinically significant feature, often reflecting a tension between the desire to die and the desire to live (45). Understanding this internal conflict can guide intervention, particularly by identifying and strengthening factors that support life-oriented motivations.

The assessment should also include a detailed reconstruction of any recent suicide attempt or self-harm episode (13). Key elements include the method used, lethality, planning, context, and the likelihood of rescue (19). These aspects provide critical information about both intent and capability, and help differentiate between impulsive and more premeditated behaviors (19).

Commonly used tools include the Columbia-Suicide Severity Rating Scale (C-SSRS) and the Ask Suicide-Screening Questions (ASQ) (26, 46, 50).

### 5.3 Risk and protective factors: beyond checklists

Although risk factors remain an important component of assessment, they should be interpreted within a broader clinical context. Internal factors such as psychiatric disorders, impulsivity, hopelessness, and prior suicide attempts interact with external variables including social support, life stressors, and access to means (8, 28).

However, focusing exclusively on risk factors may lead to an incomplete understanding of the patient’s situation (45). Protective factors—such as meaningful relationships, sense of responsibility toward others, personal values, and reasons for living—play a crucial role in modulating risk (1, 8). These elements are often underexplored in clinical practice, yet they can be decisive in moments of crisis (53).

A balanced assessment therefore requires the integration of both risk and protective factors, not as independent variables but as interacting dimensions within a dynamic system (19). This perspective shifts the focus from prediction to understanding, and from categorization to individualized formulation (34, 54).

### 5.4 Acute versus chronic risk

Distinguishing between acute and chronic suicide risk is essential for appropriate clinical decision-making. Chronic risk refers to a baseline level of vulnerability that may be associated with long-standing factors such as personality traits, psychiatric disorders, or a history of repeated self-harm (55, 56). In contrast, acute risk is characterized by a recent escalation in suicidal ideation, intent, or behavior, often in response to identifiable stressors or changes in mental state (57).

This distinction has direct implications for management. Patients with high chronic risk may not necessarily require hospitalization if acute risk is low and adequate support systems are in place (58). Conversely, even individuals without a significant prior history may require urgent intervention if acute risk is elevated. Failure to differentiate between these dimensions may lead to both over- and under-treatment.

### 5.5 The role of therapeutic engagement in assessment

Suicide risk assessment is not merely a process of information gathering but also a relational intervention. Establishing a therapeutic alliance is fundamental, particularly in emergency settings where time is limited and emotional intensity is high (22). Patients are more likely to disclose suicidal thoughts when they feel understood, respected, and not judged (5).

A collaborative and empathic approach—characterized by active listening, validation of emotional experience, and transparent communication—facilitates more accurate assessment and enhances patient engagement in subsequent care (6). Conversely, dismissive, overly directive, or purely procedural approaches may hinder disclosure and compromise both assessment and treatment.

Importantly, the assessment process itself can have therapeutic value. By helping patients articulate their distress, explore ambivalence, and identify reasons for living, clinicians may contribute to a temporary reduction in suicidal intent and open pathways for further intervention (5, 6, 59).

## 6 Management in emergency settings

### 6.1 General principles of care

The management of suicidal and self-harm behaviors in emergency settings presents unique clinical challenges (22). These environments are often characterized by time pressure, high patient turnover, and limited opportunities for longitudinal assessment (60). Nevertheless, they frequently represent the first point of contact with mental health services and therefore constitute a critical opportunity for intervention (4, 7). Although this review focuses primarily on psychiatric emergency settings, important differences exist when patients are assessed in general emergency departments.

A fundamental principle is that the management of suicidal behavior should not be approached as an isolated event but as part of a broader, ongoing treatment process (61). Even brief encounters in emergency settings can influence subsequent engagement with care and should aim to establish continuity rather than merely resolve the immediate crisis (34, 62).

Clinicians must balance the need to ensure patient safety with respect for autonomy, avoiding unnecessarily coercive measures whenever possible (24, 25). Examples include avoiding unnecessary use of physical restraints, minimizing involuntary admissions when safe alternatives exist, and prioritizing collaborative approaches. Importantly, involving the patient in decision-making processes enhances collaboration and may reduce resistance to treatment (52, 63). This participatory approach is particularly relevant in individuals with recurrent self-harm, where repeated negative experiences with healthcare systems can contribute to disengagement (64, 65).

Particular attention should be paid to patients presenting with recurrent NSSI, as repeated self-harm behaviors may reflect chronic emotional dysregulation, interpersonal difficulties, and elevated long-term suicide risk.

### 6.2 Therapeutic stance and communication

The clinician’s attitude plays a central role in the management of suicidal crises (53). A calm, non-judgmental, and supportive stance is essential for establishing trust and facilitating disclosure (66). Expressions of shock, frustration, or moral judgment may reinforce feelings of shame or invalidation and hinder therapeutic engagement (53).

Communication should prioritize empathy, validation, and active listening (25, 63). Patients should be encouraged to describe their experience in their own words, with the clinician adopting a stance of genuine curiosity and openness (52). Focusing on the “here and now” can help contain emotional distress and maintain the interaction within a manageable framework (19, 67).

Validation does not imply agreement with suicidal behavior but rather acknowledgment of the patient’s emotional suffering (68). This distinction is crucial, as it allows clinicians to maintain a supportive stance while simultaneously working toward safety and alternative coping strategies. Key principles of the clinical interview are summarized in **Table 2**.

**Table 2:** Key principles for the psychiatric emergency interview.

Recommended clinical stance (Do’s)	Counterproductive responses (Avoid)
Maintain a calm, non-judgmental, and supportive stance.	Express shock, frustration, or moral judgment.
Prioritize empathy, validation, and active listening.	Minimize the patient’s suffering or offer premature reassurance.
Adopt a stance of genuine curiosity and openness.	Focus excessively on giving advice.
Focus on the “here and now” to help contain emotional distress.	Use confrontational approaches, such as challenging intentions or issuing ultimatums.
Acknowledge and validate the patient’s emotional suffering.	Rely rigidly on protocols or checklists at the expense of relational engagement.

### 6.3 Structuring the clinical encounter

Although flexibility is required, a structured approach can enhance the effectiveness of emergency interventions (53, 66, 69). Initial contact should prioritize the establishment of a basic therapeutic connection, including clear identification of the clinician’s role and an effort to create a sense of safety (25).

Assessment and intervention should proceed in parallel rather than sequentially. While gathering clinical information, the clinician should also aim to reduce emotional intensity, enhance the patient’s sense of control, and explore potential solutions collaboratively (70). This dual process reflects the reality that, in acute settings, understanding and intervention are inseparable (63). Techniques such as grounding, validation, and structured communication may help reduce emotional intensity during assessment. Structured communication techniques may include the use of brief, clear, and emotionally validating statements, maintaining a calm tone, and organizing the interview around immediate concerns and safety.

Whenever possible, the involvement of family members or significant others should be considered, provided that the patient consents (71). Social context plays a critical role in both risk and recovery, and mobilizing available support systems can be an important component of immediate management (7).

Environmental factors should also be addressed (8). Ensuring privacy, minimizing external stressors, and reducing access to potentially harmful objects are basic but essential measures that contribute to both safety and therapeutic engagement (66).

### 6.4 Common pitfalls and counterproductive responses

Certain clinician responses may inadvertently increase distress or compromise the assessment process. Minimizing the patient’s suffering, offering premature reassurance, or focusing excessively on advice-giving can be experienced as invalidating (69). Similarly, confrontational approaches—such as challenging the patient’s intentions or issuing ultimatums—may lead to withdrawal or escalation (25).

Overreliance on protocol-driven interactions, without sufficient attention to the patient’s subjective experience, may also hinder effective management (25). While structure is important, rigid adherence to checklists at the expense of relational engagement can reduce the quality of the encounter (43, 72).

Countertransference reactions, including frustration, helplessness, or skepticism, are common in the management of self-harm and suicidal behaviors (63). Awareness of these responses is essential, as they may influence clinical judgment and interpersonal dynamics. Adopting a reflective stance allows clinicians to maintain professionalism and empathy, even in challenging situations (70).

### 6.5 From crisis containment to therapeutic engagement

The immediate goal in emergency settings is often to reduce acute risk and stabilize the situation (5, 6, 22). However, effective management extends beyond crisis containment (53). Establishing even a minimal therapeutic alliance can have significant implications for subsequent care, including adherence to follow-up and willingness to engage in treatment (63).

In this sense, the objective is not necessarily to resolve the crisis fully within the emergency setting, but rather to “create time” by reducing immediate risk and facilitating connection to ongoing care (53). This shift—from attempting to solve the problem to enabling continuity—represents a key conceptual change in the management of suicidal behavior (62, 73).

## 7 Clinical decision-making: admission versus discharge

### 7.1 The complexity of disposition decisions

Deciding whether to admit or discharge a patient presenting with suicidal or self-harm behavior is one of the most challenging aspects of emergency psychiatric care (19, 58, 72). This decision is rarely determined by a single factor and instead requires the integration of multiple clinical, contextual, and relational variables (7, 8).

Although suicide risk assessment plays a central role, disposition decisions should not be based solely on the estimated level of risk. Rather, they should reflect a broader clinical formulation that includes the patient’s mental state, the nature of the suicidal behavior, available support systems, and the feasibility of outpatient follow-up (53, 63). Overreliance on categorical thresholds of risk may lead to both unnecessary hospitalizations and unsafe discharges.

### 7.2 Indicators for hospital admission

Hospital admission may be indicated when there is a significant level of acute suicide risk that cannot be safely managed in an outpatient setting (58). This includes situations characterized by persistent suicidal ideation with intent or planning, recent suicide attempts with high lethality, or impaired judgment due to severe psychiatric conditions such as psychosis, severe mood episodes, or intoxication (19, 72).

Additional factors supporting admission include limited social support, lack of a safe environment, and inability or unwillingness to engage in outpatient care. In some cases, admission may also be required to ensure close monitoring during initiation or adjustment of pharmacological treatment (74, 75).

Importantly, the decision to admit should not be viewed solely as a response to risk, but also as an opportunity to provide a contained and structured environment in which stabilization and further assessment can occur (74, 76).

### 7.3 Criteria supporting discharge and outpatient management

Conversely, discharge with outpatient follow-up may be appropriate when acute suicide risk is low, the patient demonstrates a degree of collaboration, and adequate support systems are in place (52). Situations involving low-lethality behaviors, absence of active suicidal intent, and stable mental state may be managed safely outside the hospital setting (74).

The availability of timely follow-up care is a critical component of safe discharge (76). Patients should have access to mental health services and clear instructions regarding how to seek help in case of crisis (53). Involving family members or significant others, when appropriate, can further enhance safety and support adherence to the care plan (5, 71).

It is also important to recognize that some patients present with chronic suicidal ideation or recurrent self-harm behaviors (1). In these cases, repeated hospitalization may not only be ineffective but can sometimes reinforce maladaptive patterns (74). A careful distinction between chronic vulnerability and acute escalation is therefore essential.

### 7.4 The role of contextual and relational factors

Disposition decisions are inherently contextual. The same level of suicidal ideation may carry different implications depending on the individual’s psychosocial environment, coping resources, and prior history (38, 39, 77). Factors such as recent life stressors, interpersonal conflicts, and access to means should be considered alongside clinical variables (34).

Equally important is the patient’s capacity to engage in a collaborative plan. Agreement with the proposed management strategy, willingness to seek help if needed, and ability to adhere to recommendations are key elements in determining the feasibility of outpatient care (62). These relational aspects are often as relevant as traditional risk indicators (2).

Additional considerations may include previous patterns of crisis resolution, history of treatment engagement, impulsivity, access to lethal means, recent interpersonal losses, and the reliability of available support systems.

### 7.5 Toward a clinical decision-making framework

Rather than relying on dichotomous decisions based solely on risk categorization, a more nuanced framework is required. Clinical decision-making should integrate three core dimensions (5, 21, 23, 74):

1. Current suicide risk (including ideation, intent, planning, and recent behavior)
2. Clinical and contextual modifiers (psychiatric state, substance use, social support, environmental safety)
3. Capacity for collaboration and follow-up (engagement, insight, adherence potential)

This multidimensional approach allows for more individualized and clinically meaningful decisions, reducing the likelihood of both over- and under-intervention.

### 7.6 From risk stratification to clinical formulation

Ultimately, the decision to admit or discharge should not be understood as a binary outcome derived from risk stratification alone, but as the result of an integrated clinical formulation. This formulation incorporates both objective findings and subjective understanding, including the patient’s narrative, motivations, and ambivalence (5, 21).

In this sense, effective decision-making in emergency psychiatry requires a shift from prediction to understanding, and from protocol-driven responses to clinically informed judgment. Such an approach acknowledges the inherent uncertainty in suicide risk assessment while still providing a structured basis for action (72, 74).

**Figure 1:**
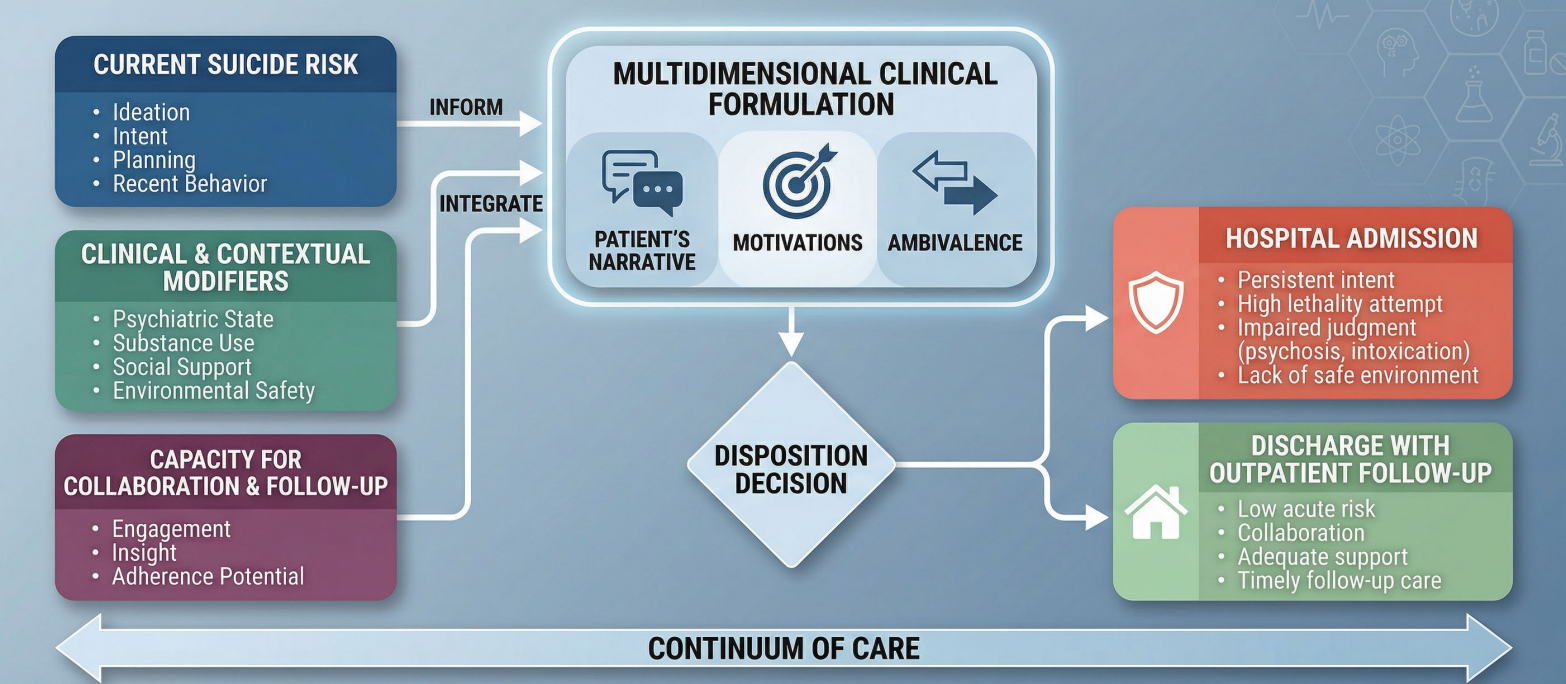
Clinical decision-making framework for disposition.

**Figure 1**. Clinical decision-making framework for the assessment and disposition of patients presenting with suicidal and self-harm behaviors in emergency settings. The model integrates three domains: current suicidal risk, clinical and contextual modifiers, and capacity for collaboration, which inform a multidimensional clinical formulation and guide disposition decisions (hospital admission vs discharge with follow-up) within a continuum of care.

## 8 Interventions

### 8.1 Non-pharmacological interventions

Non-pharmacological strategies constitute the cornerstone of intervention in patients presenting with suicidal and self-harm behaviors, particularly in emergency settings (78). These approaches aim not only to reduce immediate risk but also to address the underlying mechanisms that contribute to distress and maladaptive coping (57, 74).

A primary objective is the rapid reduction of emotional intensity and behavioral dysregulation (19, 79). This can be achieved through supportive, structured interactions that emphasize containment, validation, and collaborative problem-solving (80). Even brief interventions can be effective when they foster a sense of being understood and supported, thereby decreasing feelings of isolation and hopelessness (78).

The therapeutic relationship itself represents a key intervention. Establishing a collaborative alliance—characterized by empathy, transparency, and respect—facilitates engagement and enhances the likelihood of adherence to follow-up care (5, 22, 78). In this context, involving the patient actively in decision-making processes is essential, as it promotes autonomy and shared responsibility (81, 82).

When feasible, elements of evidence-based psychotherapeutic approaches can be incorporated into acute care (78). Interventions derived from dialectical behavior therapy (DBT), mentalization-based treatment (MBT), and other structured models have shown efficacy in reducing self-harm behaviors (83, 84). Although full implementation is not possible in emergency settings, selected techniques—such as distress tolerance strategies, emotional labeling, and perspective-taking—may be adapted to brief encounters (5, 21).

In addition, the development of a safety-oriented plan is recommended. This may include identifying warning signs, coping strategies, sources of support, and steps to take in the event of escalating distress (21, 84). Importantly, such plans should be collaboratively developed and tailored to the individual’s context (42, 43). In patients with NSSI, interventions should also explore alternative strategies for emotional regulation and distress tolerance.

### 8.2 Pharmacological considerations

Pharmacological interventions in the context of suicidal and self-harm behaviors should be guided by the underlying psychiatric condition rather than the behavior itself (84). In many cases, the acute management of self-harm does not require immediate changes in pharmacological treatment, and caution should be exercised to avoid unnecessary polypharmacy (74, 84).

When medication is indicated, the general principle is to use the lowest effective dose and to prioritize treatments with favorable safety profiles (84). Short-term use of medications may be considered to address acute symptoms such as severe anxiety, agitation, or insomnia (69). However, clinicians should remain mindful of the potential risks associated with certain drug classes, including misuse, paradoxical reactions, and toxicity in overdose (61, 85). Emerging evidence suggests that esketamine may provide rapid reduction in suicidal ideation in selected patients (86, 87).

Importantly, pharmacological strategies should be integrated within a broader treatment plan that includes psychological and social interventions (69). Medication alone is unlikely to address the complex and multifactorial nature of suicidal behavior (19, 74).

### 8.3 Integrating interventions into a continuum of care

Effective intervention in emergency settings should be understood as the initial step in a continuum of care rather than a standalone solution (21). The primary goal is to stabilize the patient, reduce immediate risk, and facilitate transition to appropriate follow-up services (22, 23).Clear communication regarding the treatment plan, including both pharmacological and non-pharmacological components, is essential (84). Continuity of care is a critical determinant of outcomes.

## 9 Clinical implications and discussion

This review proposes a clinically oriented framework that integrates risk assessment, contextual variables, and relational factors into a multidimensional formulation to guide decision-making in emergency psychiatry.

The assessment and management of suicidal and self-harm behaviors remain among the most complex tasks in psychiatric practice. Despite substantial advances in research, a persistent gap exists between empirical knowledge and its application in real-world clinical settings. This gap is particularly evident in emergency psychiatry, where decisions must often be made rapidly, under uncertainty, and with limited information.

One of the central challenges lies in the limitations of current risk prediction models (42, 43). While numerous studies have identified robust risk factors for suicidal behavior at the population level, their translation into clinically meaningful predictive tools at the individual level has been disappointing (42, 43). This has led to increasing recognition that suicide risk assessment cannot be reduced to algorithmic or checklist-based approaches. Instead, it requires a process of clinical formulation that integrates multiple sources of information, including the patient’s subjective experience, contextual factors, and temporal dynamics.

In this context, the role of clinical judgment should be reconsidered and revalued. Rather than representing a source of bias, clinical judgment—when grounded in structured assessment and reflective practice—constitutes an essential component of decision-making. The integration of empirical evidence with experiential knowledge allows clinicians to navigate complexity in ways that purely standardized approaches cannot achieve.

Another key implication concerns the importance of adopting a functional perspective on self-harm. Understanding the meanings and purposes that these behaviors serve for the individual shifts the focus from symptom suppression to targeted intervention. This approach not only enhances clinical accuracy but also fosters empathy and reduces stigmatizing attitudes, which can otherwise interfere with care.

Cultural and sociocontextual factors also influence the expression, meaning, and management of suicidal and self-harm behaviors. Help-seeking patterns, stigma, family involvement, and attitudes toward suicide may vary substantially across cultural settings, potentially affecting both assessment and intervention strategies. Future frameworks should further integrate sociocultural dimensions into emergency psychiatric care.

The findings presented in this review also highlight the central role of the therapeutic relationship, even in acute care settings. Contrary to the assumption that emergency interventions are primarily technical or procedural, relational factors—such as empathy, validation, and collaboration—play a decisive role in both assessment and management. The quality of the initial encounter may influence subsequent engagement with services and, ultimately, clinical outcomes.

From a systems perspective, the transition between emergency care and follow-up services emerges as a critical point of vulnerability. Fragmentation of care and lack of continuity may undermine the benefits of even the most well-conducted emergency intervention. Strengthening pathways of care, improving communication between services, and ensuring timely follow-up should therefore be considered priorities in suicide prevention strategies.

Emerging technologies, including digital monitoring tools, telepsychiatry, and artificial intelligence-based risk assessment models, may offer future opportunities to complement clinical decision-making. However, current evidence remains insufficient to replace individualized clinical formulation and therapeutic engagement.

Finally, this work supports a shift in focus from the prediction of suicidal behavior to its understanding and management. Given the inherent limitations of predictive models, clinical practice may benefit more from frameworks that emphasize dynamic assessment, individualized formulation, and collaborative care planning. Such an approach acknowledges uncertainty while still providing a structured and actionable basis for intervention.

### 9.1 Limitations

This review has several limitations. As a narrative and clinically oriented review, it does not follow a systematic methodology and may therefore be subject to selection bias. In addition, many recommendations discussed are influenced by the authors’ clinical experience and may not be equally applicable across different healthcare systems or resource settings. Finally, the manuscript focuses primarily on adult psychiatric emergency settings, limiting generalizability to pediatric populations and non-psychiatric emergency contexts.

## 10 Conclusions

Suicidal and self-harm behaviors represent complex and multifactorial clinical phenomena that challenge traditional models of assessment and management. In emergency psychiatric settings, where time and information are often limited, clinicians must rely on integrative approaches that combine structured evaluation with clinical judgment.

This review highlights the limitations of purely predictive models and supports a shift toward dynamic, patient-centered frameworks that prioritize understanding over categorization. Suicide risk assessment should be conceptualized not as a static determination, but as an evolving process that incorporates intent, context, ambivalence, and protective factors.

Effective management extends beyond immediate risk containment. The establishment of a therapeutic alliance, even in brief encounters, and the development of collaborative care plans are essential components of clinical practice. Decisions regarding admission or discharge should be guided by a multidimensional formulation that includes not only risk but also contextual and relational factors.

Ultimately, improving outcomes in suicidal and self-harm behaviors requires bridging the gap between evidence and practice. Emphasizing individualized care, continuity of treatment, and the central role of the clinician–patient relationship may enhance both the effectiveness and humanity of interventions in emergency psychiatry.
